# Diagnostic Accuracy of Epilepsy-dedicated MRI with Post-processing

**DOI:** 10.1007/s00062-023-01265-3

**Published:** 2023-03-01

**Authors:** Horst Urbach, Christian Scheiwe, Muskesh J. Shah, Julia M. Nakagawa, Marcel Heers, Maria Victoria San Antonio-Arce, Dirk-Matthias Altenmueller, Andreas Schulze-Bonhage, Hans-Juergen Huppertz, Theo Demerath, Soroush Doostkam

**Affiliations:** 1 Dept. of Neuroradiology, Medical Center, University of Freiburg, Breisacher Str. 64, 79106 Freiburg, Germany; 2 Dept. of Neurosurgery, Medical Center, University of Freiburg, Freiburg, Germany; 3 Dept. of Epileptology, Medical Center, University of Freiburg, Freiburg, Germany; 4 Swiss Epilepsy Centre, Klinik Lengg AG, Zurich, Switzerland; 5 Dept. of Neuropathology, Medical Center, University of Freiburg, Freiburg, Germany

**Keywords:** Epilepsy, MP2RAGE, Post-processing, MRI, Lesion

## Abstract

**Purpose:**

To evaluate the diagnostic accuracy of epilepsy-dedicated 3 Tesla MRI including post-processing by correlating MRI, histopathology, and postsurgical seizure outcomes.

**Methods:**

3 Tesla-MRI including a magnetization-prepared two rapid acquisition gradient echo (MP2RAGE) sequence for post-processing using the morphometric analysis program MAP was acquired in 116 consecutive patients with drug-resistant focal epilepsy undergoing resection surgery. The MRI, histopathology reports and postsurgical seizure outcomes were recorded from the patient’s charts.

**Results:**

The MRI and histopathology were concordant in 101 and discordant in 15 patients, 3 no hippocampal sclerosis/gliosis only lesions were missed on MRI and 1 of 28 focal cortical dysplasia (FCD) type II associated with a glial scar was considered a glial scar only on MRI. In another five patients, MRI was suggestive of FCD, the histopathology was uneventful but patients were seizure-free following surgery. The MRI and histopathology were concordant in 20 of 21 glioneuronal tumors, 6 cavernomas, and 7 glial scars. Histopathology was negative in 10 patients with temporal lobe epilepsy, 4 of them had anteroinferior meningoencephaloceles. Engel class IA outcome was reached in 71% of patients.

**Conclusion:**

The proposed MRI protocol is highly accurate. No hippocampal sclerosis/gliosis only lesions are typically MRI negative. Small MRI positive FCD can be histopathologically missed, most likely due to sampling errors resulting from insufficient harvesting of tissue.

## Introduction

Around 20–30% of patients with drug-resistant focal epilepsies are considered to be MRI-negative or non-lesional [1–6]. Non-lesional means *stricto sensu* that epileptogenic lesions are by no means made visible on MRI but have a light microscopy correlate; however, one should be aware that many consider MRI also as negative or non-lesional if a lesion was overlooked by a radiologist once.

The most prominent MRI-negative examples are focal cortical dysplasia (FCD) type I with compromise of the radial, tangential or both the radial and tangential orientation of the 6‑layered neocortex [7, 8]. The abnormal arrangement of cortical neurons in FCD type I should not be invisible on MRI as the cellular density is not changed [9]. Furthermore, the microcolumnar organization of FCD type Ia resembles neuronal radial migration streams during corticogenesis [10, 11] and may therefore result from delayed or arrested maturation at mid-gestation [12]. This also holds true for the temporal pole abnormalities associated with hippocampal sclerosis which show a reduced number of axons on diffusion microstructure imaging [13]. The U‑fiber layer beneath FCD type I often contains an excessive number of heterotopic neurons leading to a blurring of the gray-white matter junction. These displaced neurons form complex synaptic plexus within the U‑fiber layer, some axons of which ascend into the cortex to be integrated into synaptic networks [14]. Accordingly, FCD type I is often accompanied by a regional reduction (lobar hypoplasia/atrophy) and a mild signal increase of the white matter (blurring) making them indirectly visible.

Other examples of MRI-negative histopathological specimens are Palmini et al.’s mild malformations of cortical development (mMCD); however, at least mild malformation of cortical development with excessive white matter neurons (mMCD type 2) produces a blurring of the gray-white matter junction on T2-weighted/FLAIR (Fluid attenuated inversion recovery) sequences [15]. The difficult distinction between no lesion, FCD type I, and mMCD is indicated by the low intrarater agreement documented in a blinded classification of 26 specimens by 8 neuropathologists [16].

Astrogliosis without neuronal loss, called no hippocampal sclerosis, gliosis only may also be missed on MRI. It has been described post-mortem [17] and in surgical specimens [18]. Compared to classical hippocampal sclerosis which accounts for at least 95% of surgical cases, MRI shows absence of pronounced volume loss, only slight T2-weighted signal increase and bilaterality of the disease [19–21].

Subtle epileptogenic lesions, however, can also be overlooked due to a low MRI quality (MRI false negative). A retrospective evaluation of the MRI-negative cases from the Bonn series 2000–2006 revealed that 1/3 of epileptogenic lesions were overlooked [2]. With higher field strength and epilepsy-dedicated sequences aiming at enhancing the visibility of FCD by suppressing the white matter and CSF signal (FLAWS), nulling the signal of voxel containing gray and white matter (EDGE), or enhancing the B1 homogeneity (MP2RAGE) the rate of overlooked lesions can be reduced [22–27]. The standardized use of a post-processing tool may enhance the detection rate by around 30–41% or even 78% [28–35].

In order to evaluate the accuracy of such an epilepsy-dedicated MRI including post-processing we correlated MRI, histopathology and postsurgical seizure outcomes in a consecutive series of patients with drug-resistant focal epilepsies undergoing resective epilepsy surgery.

## Material and Methods

### Patients

A surgical data base including patients with drug-resistant focal epilepsy undergoing surgery was retrospectively analyzed over a 3-year period. Inclusion criteria were as follows:

- Epilepsy-dedicated MRI on a Siemens 3 T Prisma scanner (Siemens Healthineers, Erlangen, Germany) including a MP2RAGE sequence.
- Resective surgery, such as anterior temporal lobectomy, amygdala-hippocampectomy and (extended) lesionectomy.
- Post-processing using the Morphometric Analysis Program (MAP 18).

Other presurgical evaluations such as video-EEG, stereo electroencephalography (SEEG), and ^18^F FDG-PET were considered if available. Patients who had had prior surgery were excluded.

### MRI Protocol

The MRI protocol was similar to the International League against Epilepsy (ILAE) recommended harmonized neuroimaging of epilepsy structural sequences (HARNESS) MRI protocol [36]. In addition, a sagittal MP2RAGE sequence was acquired. The study was approved by the local ethics committee (123/20) (Table 1).

**Table 1:** Epilepsy-dedicated MRI protocol (3 T Magnetom Prisma, Siemens Healthineers, Erlangen, Germany)

MRI sequence	No. of slices/thickness (mm)	Voxel size (mm^3^)	TI/TR/TE/α (ms/ms/ms/°)	Acquisition time (min:s)
Sag 3D MPRAGE	160/1	1 × 1 × 1	900/2000/2.26/12	4:40
Sag 3D FLAIR-SPACE	160/1	1 × 1 × 1	1800/5000/388/var	6:52
Ax 2D T2-TSE	42/3	0.4 × 0.4 × 3	5040/102/150	4:34
Ax 2D T2*	23/5	0.7 × 0.7 × 5	639/19.9/20	2:33
Cor 2D T2-STIR	40/2	0.4 × 0.4 × 2	100/5390/25/140	8:07
Cor 2D FLAIR	68/2	0.7 × 0.7 × 2	2500/9000/87/150	4:14
Ax 2D DWI-SE EPI	23/5	0.6 × 0.6 × 5	3400/85	0:46
Sag 3D MP2RAGE	192/1	1 × 1 × 1	700, 5000/2000/2.9/4	8:52

*MPRAGE* Magnetization Prepared Rapid Gradient Echo, *FLAIR SPACE* Fluid-Attenuated Inversion Recovery—Sampling Perfection with Application-optimized Contrasts by using flip angle Evolution, *TSE* Turbo Spin Echo, *STIR* Short Tau Inversion Recovery, *DWI* Diffusion-Weighted Imaging, *SE* Spin Echo, *EPI* Echo Planar Imaging, *TI* inversion time, *TR* repetition time, *TE* echo time, *α* flip angle, *var* variable flip angle, *Sag* sagittal, *Ax* axial, *Cor* coronar, T2* T2 gradient echo

### Post-processing

The unified images of the MP2RAGE sequence were processed with SPM 12 (http://www.fil.ion.ucl.ac.uk/spm/) running in MATLAB R2014b (MathWorks, Natick, MA, USA). In a first step, DICOM images were converted to Neuroimaging Informatics Technology Initiative (NIfTI) format. NIfTI images were segmented into gray matter (GM), white matter (WM) and cerebrospinal fluid (CSF) maps and normalized to the Montreal Neurological Institute (MNI) space. Using the MAP18 software, junction, extension, and thickness images were calculated as described before [37, 38]. In addition, these morphometric maps were used as input for an artificial neural network (ANN) trained with MRI data of FCD patients and healthy controls as described elsewhere [39]; however, it should be noted that this ANN has been trained with MPRAGE data as FCD cases with MP2RAGE sequences required for such training are currently not available in sufficient quantity. The output of the ANN after classification of all voxels in the unified MP2RAGE image comprises a FCD probability map with values closer to 1 indicating voxels more likely to be dysplastic tissue and values closer to 0 representing non-dysplastic brain tissue or compartments outside of the brain. Finally, all results of MRI post-processing (i.e., morphometric maps and FCD probability map) were inversely normalized (i.e., transferred to native space), the FCD probability maps were co-registered to the MP2RAGE unified images, reconverted to Digital Imaging and Communications in Medicine (DICOM) format and exported to the picture archiving and communication system system (PACS) [27].

### Analysis

The MRI reports were retrieved from the PACS system (Dedalus Deep Unity Diagnostic, Bonn, Germany). Histopathology and outcome reports according to Engel et al. and the International League Against Epilepsy (ILAE) [40, 41] were retrieved from the clinical information system system (MeDoc, University Medical Center Freiburg, Germany). Invasive presurgical work-up via stereotactically placed electrodes for SEEG recordings were considered when necessary. Discordant MRI and histopathology findings were reviewed by a neuroradiologist (HU) and a neuropathologist (SD).

## Results

**Fig. 1:**
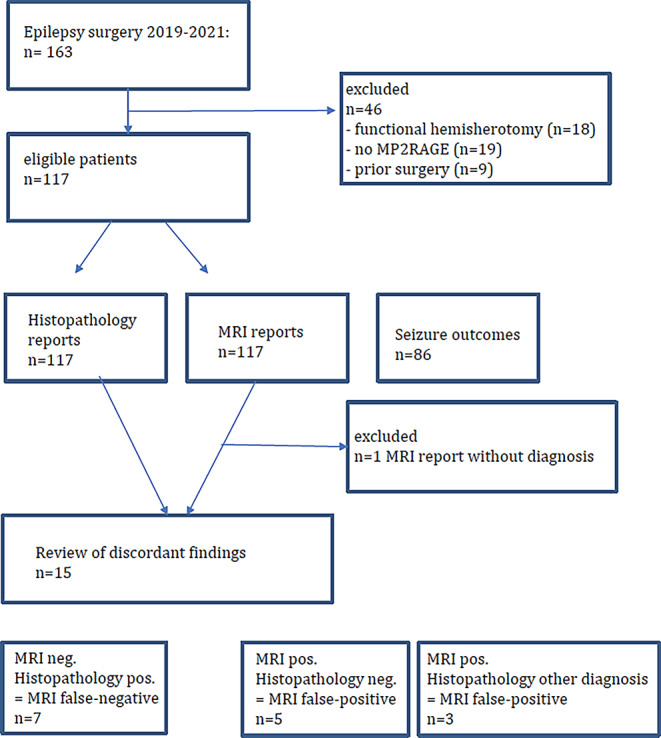
Study flowchart

A total of 116 patients (64 male, 52 female) were included in the analysis (Fig. 1). Mean age at surgery was 26 years (8 months–76 years of age). The histopathologically defined lesions can be categorized into 6 groups [44]: malformations of cortical development, hippocampal sclerosis, long-term epilepsy-associated tumors (LEAT), vascular malformations, glial scars, no lesions (Table 2).

**Table 2:** Synopsis of histopathology and MRI for the subgroups malformations of cortical development, hippocampal sclerosis, long-term epilepsy-associated tumors (LEAT), vascular malformations, glial scars, and no lesion. The distribution is similar to the neuropathological series of the European Epilepsy Brain Bank consortium including 9523 patients from 12 European countries [44]. Outcome data were available in 86 patients: The percentages of Engel class IA outcome are related to the histopathology findings

Lesion type	Histopathology	MRI	Outcome/Engel IA (histopathology)
Malformations of cortical development	FCD II: *n* = 28	FCD and mMCD: *n* = 38	17/23 (74%)
	mMCD: *n* = 6		2/5 (40%)
Hippocampal sclerosis	Isolated HS: *n* = 14	*n* = 17	5/8 (63%)
	HS with anterior temporal lobe abnormalities (gray white matter blurring, FCD IIa, FCD IIIa, mMCD): *n* = 16	*n* = 14	9/11 (82%)
	No hippocampal sclerosis/gliosis only: *n* = 3	*n* = 0	3/3 (100%)
LEAT	Ganglioglioma: *n* = 14	*n* = 15	6/8 (75%)
	DNT: *n* = 5	*n* = 5	3/3 (100%)
	PXA: *n* = 2	*n* = 2	0
	PLNTY: *n* = 1	*n* = 0	0
Vascular malformations	Cavernoma: *n* = 6	*n* = 6	5/6 (83%)
Glial scars	Scar: *n* = 6	*n* = 7	4/5 (80%)
No lesion	Negative: *n* = 15	Negative: *n* = 8 temporal lobe meningo-encephaloceles: *n* = 4	7/14 (50%)

*FCD* focal cortical dysplasia, *mMCD* mild malformation of cortical development, *HS* hippocampal sclerosis, *DNT* dysembryoplastic neuroepithelial tumor, *PXA* pleomorphic xanthoastrocytoma, *PLNTY* polymorphous low grade neuroepitheilai tumor of the young

## Malformations of Cortical Development

**Fig. 2:**
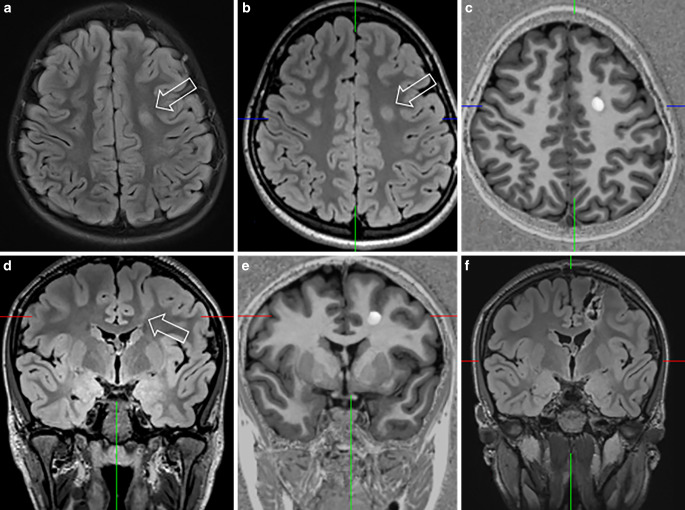
A 12-year-old girl presented with daily sensory and bilateral tonic seizures since the age of 6 years. An MRI at the age of 10 years was considered normal (**a**: *arrow*). MRI at the age of 11 years revealed a FCD in the depth of the left superior frontal sulcus (**b**–**e**) with a thickened cortex and a transmantle sign (**d**: *arrow*). Post-processing of the MP2RAGE images using the MAP tool highlights the lesion histopathologically characterized as FCD type IIB (**c**, **e**). MRI after 3 months shows the resection of the FCD (**f**). Outcome after 6 months was Engel 1B

**Fig. 3:**
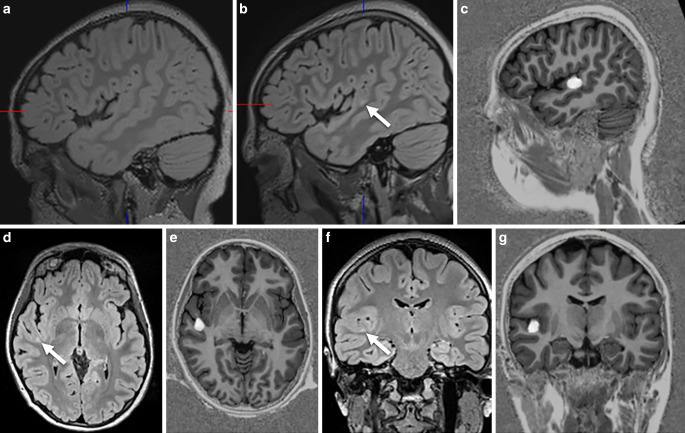
A 14-year-old girl presented with sleep-related, hyperkinetic, tonic and clonic seizures associated with fear since the age of 5. MRI at the age of 5 did not disclose a lesion (**a**). MRI at the age of 13 showed a slight FLAIR hyperintensity at the gray-white matter border (**b**,**d**,**f**: *arrow*) highlighted by post-processing of MP2RAGE images using the MAP tool (**e**,**g**)

**Fig. 4:**
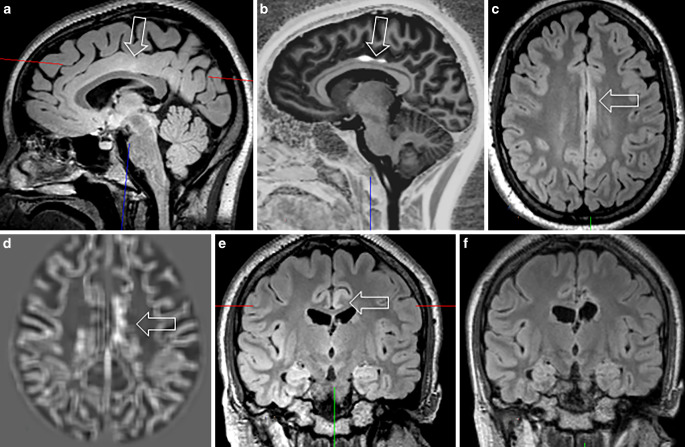
A 14-year-old girl presented with hypermotor seizures since the age of 11. MRI showed a hyperintense left-sided cingulate cortex (**a**,**c**,**e**: *arrow*). The lesion was found by scrolling through the co-registered MP2RAGE images (**b**: *arrow*). The junction parameter map displays the gray-white matter blurring (**d**: *arrow*). Histopathology revealed gliotic changes, a gray-white matter blurring, some ectopic white matter neurons, no dysplastic neurons, no balloon cells. MRI after 3 months confirmed that the lesion was resected (**f**). The patient was seizure-free 12 months following surgery

**Fig. 5:**
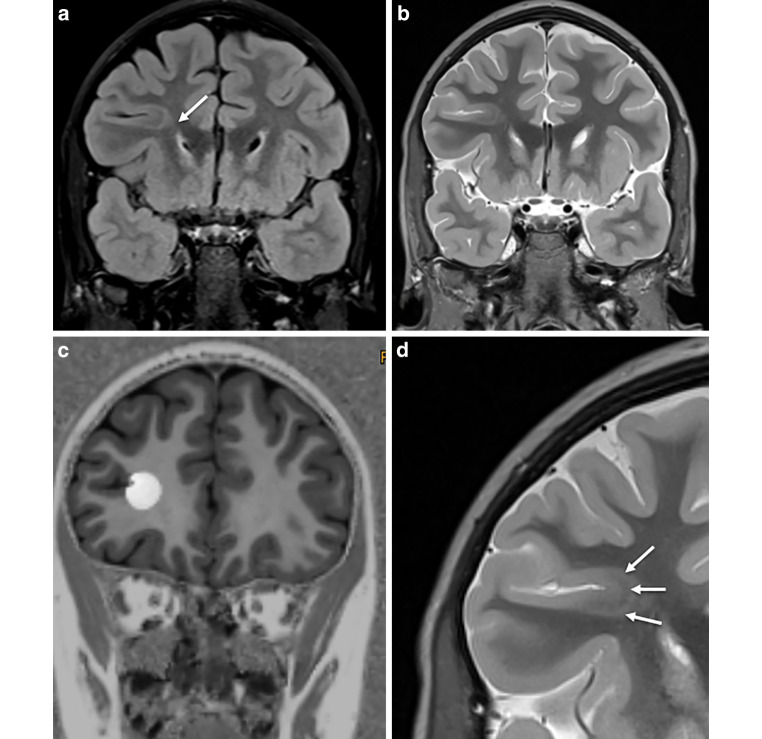
FCD type IIA in the depth of the right inferior frontal sulcus in a 5-year-old girl with focal motor, tonic and atonic seizures. The FCD shows a funnel-shaped hyperintensity tapering towards the right frontal horn (visible on FLAIR (**a**) and on T2-weighted (**b**) images: *arrow*). The initial histopathological diagnosis of a multinodular vacuolating tumor was revised due to the characteristic MRI pattern also highlighted by post-processing (**c**). Furthermore, the intracortical black sign is described as a pathognomonic feature of a FCD IIB (**d**: *arrows*)

**Fig. 6:**
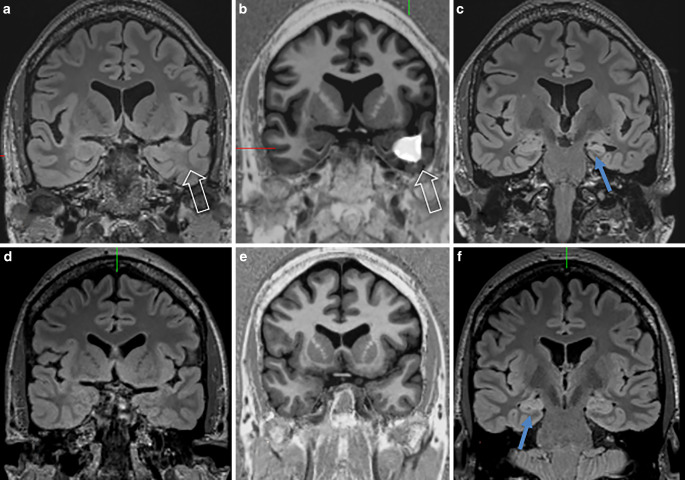
Left-sided hippocampal sclerosis ILAE type 1 (**c**: *blue* *arrow*) with associated gray-white matter blurring (**a**,**b**: *hollow arrows*) in a 38-year-old patient who had fever-related seizures at the age of 8 months. Right-sided hippocampal sclerosis ILAE type 1 (**f**: *blue arrow*) in a 50-year-old patient with temporal lobe seizures since the age of 13 years. Coronal FLAIR (**d**) and post-processed MP2RAGE images (**e**) showed no associated gray-white matter blurrin*g*

The MRI detected abnormalities suggestive of a FCD type II (increased cortical thickness, blurring of the gray-white matter junction, transmantle sign, abnormal gyral/sulcal pattern) or of a mMCD type II (blurring of the gray-white matter junction) in 38 patients (33%) ([15, 42]; Figs. 2, 3, 4, 5 and 6).

On histopathology, a FCD was found in 28 patients and an mMCD type II in 6 patients. In 1 of the 28 patients the FCD was associated with a glial scar, 5 patients without histopathologic abnormalities showed clear MRI abnormalities which were also appreciated on the fully automatic MAP analysis (Fig. 4) and became seizure-free following surgery. Thus, the most likely explanation for missing the histopathological diagnosis in these five patients was a sampling error. Of interest, one FCD type II with an intracortical black line sign was confirmed as FCD type IIA in a second microscopic round after additional resected tissue was considered ([43]; Fig. 5). Overall, 22 patients with FCD type II had earlier MRI scans stored in the PACS and written reports stored in the medical records: 13 FCD type II (59%) were overlooked (Fig. 2) or not visible at all (Fig. 3).

## Hippocampal Sclerosis

Of the patients 31 had hippocampal sclerosis on MRI: in 17 patients, hippocampal sclerosis was the only finding, in 14 patients MRI reports noted additional anterior temporal lobe abnormalities which were described as gray-white matter blurring. Histopathology reports described 14 isolated hippocampal sclerosis and 16 patients with additional temporal lobe abnormalities described as a blurry gray-white matter border (*n* = 13), FCD type IIA (*n* = 1), FCD type IIIa (*n* = 1) or mMCD (*n* = 1) (Fig. 6).

A hippocampal sclerosis confined to the hippocampal head on MRI was considered negative on histopathology.

A total of three patients were MRI-negative but had histopathologically no hippocampal sclerosis/gliosis only [19]. They were successfully operated on after SEEG electrodes showed ictal spikes in the hippocampus (Engel IA after 12 months).

## Long-term Epilepsy-Associated Tumors (LEAT)

A total of 22 patients (19%) had long-term epilepsy-associated tumors (LEAT) of which 14 gangliogliomas and 5 dysembryoplastic neuroepithelial tumors (DNT) were identified on MRI due to the cortical/subcortical cystic elements. The DNTs were separated from gangliogliomas via the multicystic appearance of the glioneuronal element and two pleomorphic xanthoastrocytomas (PXA) were identified due the meningocerebral contrast enhancement. The polymorphous low-grade neuroepithelial tumor of the young (PLNTY) was considered to be most likely a ganglioglioma, although it had no cystic elements and a stalk-like extension into the white matter.

## Vascular Malformations, Glial Scars

Of the patients six had cavernomas with hemosiderin staining of the brain parenchyma on T2* images, and seven patients had cortical scars. One glial scar on MRI was considered to have an associated FCD IIA on histopathology.

## No Lesions

Histopathology was negative in 10 patients suffering from temporal lobe epilepsy (Table 2) and 4 of the 10 patients were operated on for temporal lobe meningoencephaloceles, so light-microscopic abnormalities were not expected. One had a hippocampal sclerosis on MRI which was not proven by histopathology. With respect to the remaining five patients, all of them were operated on after SEEG electrodes showed seizure onset within the hippocampus and/or the anterior temporal lobe. Only one of them was seizure-free (Engel IA) 12 months following surgery.

In summary, MRI and histopathology were concordant in 101 and discordant in 15 patients. If we consider histopathology as gold standard, sensitivity of MRI is 92.8%, specificity 57.8%, and accuracy 87%; however, we would disregard the fact that five suspected FCD with unequivocal MRI findings which were not confirmed by histopathology and therefore counted as false positives were likely FCD as the patients were seizure-free following surgery (Figs. 4 and 5).

## Discussion

Within a 3-year period, we encountered three MRI-negative, histopathology-positive patients undergoing epilepsy surgery: all had no hippocampal sclerosis/gliosis only on histopathology and were successfully operated on after SEEG recordings showed ictal discharges in the hippocampus; however, we also identified six patients with both visible and fully automatically detected MRI lesions that did not have a histopathology substrate. As the five patients with MRI lesions suggestive of a FCD or mMCD (e.g. Figs. 4 and 5) were seizure-free following surgery it is very likely that the small lesions were missed due to sampling errors.

The rate of MRI-true negative drug-resistant focal epilepsy is thus distinctly lower than the reported range of 10–30% [1–6].

One reason might be that a considerable portion of MRIs are false negative because FCDs are poorly detectable and thus are overlooked. This is especially true for small FCD in the depth of a sulcus. The detection of such lesions is facilitated by high quality MRI scans in conjunction with MRI post-processing, e.g., by means of morphometric analysis. To this end, the Morphometric Analysis Program (MAP18) is employed in our center, which was specifically trained to identify abnormalities such as an abnormal cortical thickness or extension or a blurring of the gray-white matter junction which are summarized in a so-called FCD probability map. The MAP tool distinctly benefits from MP2RAGE images which display the FCD with higher z‑scores and larger volumes [25, 26]. When the FCD probability maps are inversely normalized (i.e., transferred to native space), co-registered to the original MP2RAGE image, reconverted to DICOM format and exported to the PACS system the FCD can be found very quickly (“within 1 minute”) by scrolling through the co-registered data set [27].

The high rate of epilepsy initially considered to be MRI-negative underlines the need to post-process structural MRI scans. Fully automatic MRI analysis may also help to gain confidence that a subtle lesion is a FCD and to proceed with invasive evaluations such as SEEG or directly with surgery.

While hippocampal sclerosis is also clearly visible on MRI, uncertainty exists about the relevance of no hippocampal sclerosis/gliosis only and the additional finding of a gray-white matter blurring of the anterior temporal lobe. There are hints from microscopic examinations and diffusion microstructure imaging that the blurring next to hippocampal sclerosis is a maturation disorder with fewer axons in the anterior temporal lobe [13, 45]. This constellation is more likely when the anterior temporal lobe is smaller than its counterpart and clinically supported by the fact that most patients with gray-white matter blurring associated with hippocampal sclerosis had the precipitating event leading to hippocampal sclerosis before the age of 2 years [46]; however, a FCD type I, a FCD type IIa, and a mMCD type II were also described in three patients with gray-white matter blurring underlining that MRI is capable of detecting abnormalities but cannot separate between these different histopathological diagnosis [16].

A total of 10 histopathology-negative patients suffered from temporal lobe epilepsies, 1 had hippocampal sclerosis on MRI, 4 had antero-inferior meningoencephaloceles and 5 were completely MRI-negative. In these patients, epileptogenicity is typically proven via SEEG recordings, in this series only one of five patients became seizure-free.

## Limitations

A study to determine the real sensitivity, specificity and accuracy of MRI in epilepsy imaging would randomly include focal epilepsy patients with the problem being that there is no ground truth (histopathology after surgery). This selection bias is reflected in this series by the fact that patients with a MRI lesion more often underwent surgery than those without; however, the only way to prove whether a MRI lesion has a morphological substrate is to relate it to histopathology. The finding that all MRI-negative and histopathology-negative patients had temporal lobe epilepsies and underwent anterior temporal lobectomy could be due to the fact that is easier to locate an epilepsy syndrome to the anterior temporal lobe than to the other brain lobes, and that patients less frequently have an overlap of the epileptogenic zone with eloquent cortex compared to the central, parietal or frontal neocortex.

## Conclusion

True MRI-negative epilepsy defined as light-microscopic abnormalities not being visible on MRI are rare and not in the reported range of 10–30%.

Subtle FCD can be easily missed and represent a major subgroup of MRI false negative epilepsy. They may be highlighted with post-processing tools such as the Morphometric Analysis Program (MAP).

Subtle FCD can even be histopathologically missed. In this constellation, the gold standard for a correct MRI-based diagnosis should be seizure freedom of the patient.

MRI-negative is a difficult term as it includes overlooked lesions, typically FCD type II in the depth of a sulcus. There were three true MRI-negative cases in this surgical series who had no hippocampal sclerosis/gliosis only on histopathology acknowledging the bias that the chance for being operated on is higher when MRI shows a typically epileptogenic lesion.
